# Polyphenols as Food Supplement Improved Food Consumption and Longevity of Honey Bees (*Apis mellifera*) Intoxicated by Pesticide Thiacloprid

**DOI:** 10.3390/insects12070572

**Published:** 2021-06-23

**Authors:** Marian Hýbl, Petr Mráz, Jan Šipoš, Irena Hoštičková, Andrea Bohatá, Vladislav Čurn, Tomáš Kopec

**Affiliations:** 1 Department of Zoology, Fisheries, Hydrobiology and Apiculture, Faculty of AgriSciences, Mendel University in Brno, Zemedelska 1, 613 00 Brno, Czech Republic; mario.eko@seznam.cz (M.H.); jan.sipos@mendelu.cz (J.Š.); 2 Department of Genetics and Agricultural Biotechnology, Faculty of Agriculture, University of South Bohemia in Ceske Budejovice, Studentska 1668, 370 05 Ceske Budejovice, Czech Republic; mrazpe01@zf.jcu.cz (P.M.); jelini00@zf.jcu.cz (I.H.); curn@zf.jcu.cz (V.Č.); 3 Department of Crop Production, Faculty of Agriculture, University of South Bohemia in Ceske Budejovice, Studentska 1668, 370 05 Ceske Budejovice, Czech Republic; bohata@zf.jcu.cz; 4 Department of Animal Breeding, Faculty of AgriSciences, Mendel University in Brno, Zemedelska 1, 613 00 Brno, Czech Republic

**Keywords:** cage experiments, cytochrome P450, detoxification, food intake, mortality rate

## Abstract

**Simple Summary:**

Worldwide, mass losses of honey bee colonies are being observed more frequently. Poor nutrition may cause honey bees to be more susceptible to pesticides and more vulnerable to diseases, and as a direct result of this, honey bee colonies can collapse. Another cause of mass bee colony collapse that is no less important is the use of pesticides. The level of toxicity of most pesticides is greatly affected by nutrient uptake. In addition, the honey bee genome is known to be specific for a significantly lower number of genes associated with detoxification compared with other insect species. Intake of phenolic and flavonoid substances in food can lead to increased expression of genes encoding detoxification enzymes in bees. Therefore, in this study, we evaluated in vitro the effect of phenolic and flavonoid substances on bee mortality and food consumption in the case of intoxication by pesticide thiacloprid. The results of this study showed a significant positive effect on honey bee survival rate as well as increased food intake. In addition, the expression level of genes encoding detoxification enzymes was determined.

**Abstract:**

Malnutrition is one of the main problems related to the global mass collapse of honey bee colonies, because in honey bees, malnutrition is associated with deterioration of the immune system and increased pesticide susceptibility. Another important cause of mass bee colonies losses is the use of pesticides. Therefore, the goal of this study was to verify the influence of polyphenols on longevity, food consumption, and cytochrome P450 gene expression in worker bees intoxicated by thiacloprid. The tests were carried out in vitro under artificial conditions (caged bees). A conclusively lower mortality rate and, in parallel, a higher average food intake, were observed in intoxicated bees treated using a mixture of phenolic acids and flavonoids compared to untreated intoxicated bees. This was probably caused by increased detoxification capacity caused by increased expression level of genes encoding the cytochrome P450 enzyme in the bees. Therefore, the addition of polyphenols into bee nutrition is probably able to positively affect the detoxification capacity of bees, which is often reduced by the impact of malnutrition resulting from degradation of the environment and common beekeeping management.

## 1. Introduction

One of the most worrying phenomena is the global mass losses of honey bee colonies, including in Europe and the USA [1,2]. Along with diseases, nutrition stress and malnutrition appears to be one of the main causes of bee mortality [3,4,5]. The healthy development and survival of bee colonies depends to a large extent on the availability and quality of nutrients in the environment [3,6]. However, the availability and diversity of bee food resources are steadily declining due to the ever-increasing intensification of agriculture and the associated changes in the landscape, leading to a decrease in environmental sustainability [7]. As a result, there has been a decrease in the diversity of flowering plants, and low species diversity of blooming plants means reduced availability and diversity of macro- and microelements in bee nutrition [4,8], which, in the end, negatively affects bee populations [7,9]. The lack of nutrients is also the result of inefficient beekeeping practices; when replenishing winter supplies, bees are often provided only with a solution of sugar and artificial pollen substitutes. These food supplements usually lack nutrients that are naturally occurring in bees’ natural diets [10]. Consequently, bee colonies are not provided with full-value nutrition [11]. Poor nutrition may cause greater susceptibility to pesticides [12], more vulnerability to diseases [13], and, as a direct result of this, the number of honey bee colonies may be decreasing [14].

Another cause of mass bee colony collapse that is no less important is the use of pesticides [15,16], which can act synergistically with other pesticides [17] or with pathogens [18,19]. The level of toxicity of most pesticides varies depending on many factors, including the means of exposure, the age of the bees, the fitness of the colonies or bee subspecies [20,21], and the optimal nutrient distribution [4,22]. In addition, the degree of toxicity of different pesticides may vary depending on whether they are tested on individual bees or on whole colonies. In vitro tests often show high pesticide toxicity and associated negative effects on bees [17,18,23]; in contrast, entire colonies appear to be relatively less susceptible to pesticides [24]. A similar trend can be observed in other social bees such as bumblebees [25]. In a broader context, some bee species may even be advantaged in anthropogenic areas such as agricultural land or urban areas [26]. However, the results of the study by Alburaka et al. [27] suggest that, while neonicotinoids do not directly affect the health and strength of bee colonies, they indirectly weaken bee health by inducing physiological stress and increasing the burden of pathogens.

However, the bee genome is known to be specific for a significantly lower number of genes associated with detoxification compared to other insect species. Where the honey bee has only 46 genes encoding the cytochrome P450 enzyme, which is thought to be the major enzyme responsible for detoxification, other insect species have around 80 or more genes encoding the cytochrome P450 enzyme [28]. There are several honey bee cytochrome P450 genes that have defined functions, including CYP9Q1, CYP9Q2, and CYP9Q3. These genes metabolize both natural and synthetic xenobiotics [29].

The intake of phenolic and flavonoid substances, which are commonly found in honey and, to a greater extent, in pollen, via food can lead to an increased expression of genes encoding cytochrome P450 enzyme in bees. The amount and proportion can vary significantly depending on food sources [30]. Of these, the highest efficacies have been observed for p-coumaric acid and quercetin [31]. The natural diet of bees usually contains a large amount and great diversity of phenolic acids, flavonoids and their derivatives [32,33] and it is their different amounts and proportions that influence the detoxifying effects [31].

The first goal of this study was to determine the real effect of phenolic acids and flavonoids in vitro on the mortality of bees intoxicated by thiacloprid, one of the most widely used neonicotinoids. The second aim was to determine the effect of phenolic substances on the rate of food intake by bees; and the last target, although no less important, was to determine the expression level of several genes potentially responsible for detoxification via the enzyme cytochrome P450.

## 2. Materials and Methods

The experiment was carried out at the beginning of the summer of 2019 in Brno (South Moravia, Czech Republic)

### 2.1. Bees

The honey bees used in this study were obtained from the experimental apiary of Mendel University in Brno. Honey bees from four colonies were used (one frame with hatching bees per colony). The colonies were maintained following standard beekeeping practices. In all the bee colonies, inseminated queens belonging to *Apis mellifera carnica* were used. As a result, the genetic variability of bees in individual colonies was reduced so that the average coefficient of relatedness between workers from one colony was r = 0.5.

The brood frames with hatching bees (one from each colony) were incubated at 35 °C and 65–80% relative humidity for 12 h. This allowed bees of the same age ± 12 h to be obtained. Then the frames were brushed, and all bees were mixed together and divided into four groups according to the treatment with three replications (three cages each). There were 40 bees of the same age in each cage. The cages were maintained for 2 weeks in the thermostat with conditions 30 °C and 65–70% relative humidity [34]. The bee mortality and food consumption were noted down every day and dead bees were continuously removed from the cages.

### 2.2. Chemicals

The sucrose solution consisted of 50% (*w/v*) sucrose and distilled water. A dosage of thiacloprid was mixed with the sucrose solution in two different concentrations, 35 mL/L or 70 mg/L, depending on the treatment [19].

The mixture of phenolic compounds consisted of 200 mg/kg of phenolic acids and 10 mg/kg of flavonoids in proportions based on the real concentrations found in common honey [33]. The concentration of p-Coumaric acid was scaled up on the basis of Mao et al. [30]. The final contain of phenolic compounds is in Table 1. The thiacloprid and sucrose were purchased from Sigma Aldrich (Schnelldorf, BO, Germany), phenolic acids and flavonoids were purchased from Alfa Aestar (Kandel, RP, Germany).

### 2.3. Design of the Experiment

The bees in cages were fed with two top feeders with scales (*ad libitum*) per cage, enabling measurement of the daily food consumption. The rate of consumption of a prequantified amount by a set number of live bees was evaluated over a set time period. The experimental groups were set up as follows:

1. Treatment TL—sucrose solution with a low dosage of Thiacloprid (35 mg/L).
2. Treatment FTL—sucrose solution (50% *w/v*) with a mixture of phenolic compounds and low dosage of Thiacloprid (35 mg/L).
3. Treatment TH—sucrose solution (50% *w/v*) with high dosage of Thiacloprid (70 mg/L).
4. Treatment FTH—sucrose solution (50% *w/v*) with a mixture of phenolic compounds and a high dosage of Thiacloprid (70 mg/L).
5. Treatment F—sucrose solution (50% *w/v*) and a mixture of phenolic compounds.
6. Treatment C—sucrose solution (50% *w/v*).

### 2.4. RNA Isolation and RT-qPCR

Samples for studying gene expression were collected as a bulk of three bees and frozen immediately in liquid nitrogen, and were stored at −80 °C. Total RNA was extracted using the TRI Reagent (MRC, Montgomery, OH, USA) according to manufacturer’s instructions. Contaminating DNA was removed using the DNA-freeTMKit (Ambion, supplied by ThermoFisher scientific, Loughborough, UK). BioSpec Nano (Shimadzu, Nakagyo-ku, Kyoto, Japan) was used to quantify RNA (OD260) and to assess sufficient quality (OD260/280 ratio and OD260/230 ratio). cDNA templates were prepared using a Standard Reverse Transcription Protocol (Promega, Madison, WI, USA) and stored at −20 °C until use.

The RT-qPCR was performed on the QuantStudio™ 6 Flex Real-Time PCR System (Applied Biosystems, supplied by ThermoFisher scientific, Loughborough, UK) using Power SYBR^®^ Green PCR Master Mix (Applied Biosystems, supplied by ThermoFisher scientific, Loughborough, UK) in a 96-well reaction plate using parameters recommended by the manufacturer (2 min at 50 °C, 10 min at 95 °C and 40 cycles of 15 s 95 °C, 1 min of 60 °C, 15 s at 95 °C, 1 min at 60 °C and 15 s at 95 °C). The three replicates and no-template controls were included. The specificity of amplification was determined by dissociation curve analyses. A comparative threshold cycle method was applied to determine relative concentrations of mRNA. The primers used are shown in Table 2. All the gene expression levels were normalized to Am Rp49 gene expression, as a reference gene [35], and the obtained data were normalized to Am Rp49 using the ΔΔCT method according to Livak, Schmittgen [36].

### 2.5. Data Analyses

The survival curves were fitted by the Kaplan-Meier method. On the basis of this method, the survival probability for each tested treatment during 14 days of observation was estimated [40]. The conclusive difference between each survival curve was evaluated by log-rank test [41]. The log-rank test compares a monitored case number with the case number that would have been expected under the null hypothesis (i.e., identical survival curves). All data were analyzed using the R statistical program (R Core Team, 2017).

Daily food intake was analyzed using the statistical program Statistica 12. The effect of fed substances on the rate of diet consumption was tested by the analysis of variance procedure ANOVA (post hoc analysis using Tukey test), preceded by a normality test. Statistical significance was tested at a level of significance α = 0.05.

## 3. Results

**Figure 1:**
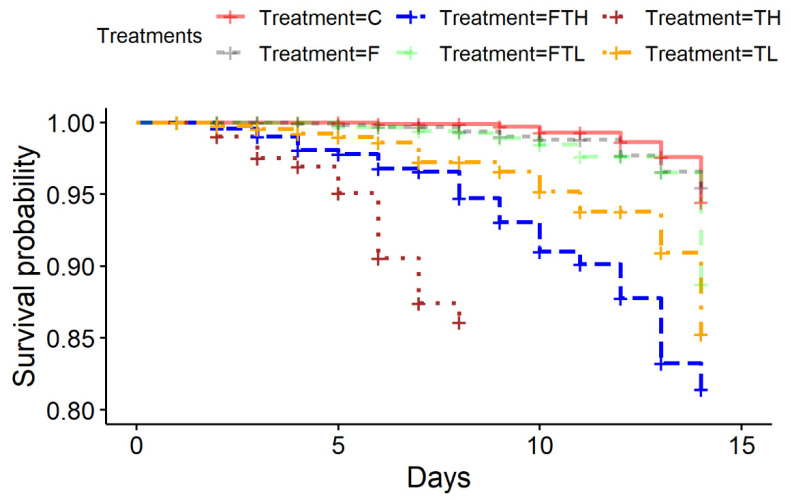
The relationship between the bee mortality and the treatments over 14 days (Kaplan-Maier survival analyses). Legend: TH—sucrose solution (50% *w/v*) with high dosage of Thiacloprid (70 mg/L), FTH—sucrose solution (50% *w/v*) with a mixture of phenolic compounds and a high dosage of Thiacloprid (70 mg/L), TL—sucrose solution with a low dosage of Thiacloprid (35 mg/L), FTL—sucrose solution (50 *w/v*) with a mixture of phenolic compounds and low dosage of Thiacloprid (35 mg/L), F—sucrose solution (50% *w/v*) and a mixture of phenolic compounds, C—sucrose solution (50% *w/v*).

The bee survival rate corresponding fed treatment is presented in Table 3 and Figure 1. The FTH group exhibited a significantly lower mortality rate than group TH (*p* < 0.001), but a higher mortality rate than the control groups C and F (*p* < 0.001 for both). Comparatively lower mortality rates were observed in the treatment FTL than in the treatment TL (*p* < 0.001), although the mortality rate was higher than in the control group C (*p* = 0.03), but no significant differences were observed in comparison with group F (*p* = 0.17). Additionally, no significant differences were registered between control groups C and F (*p* = 0.44). Significant differences were also observed between TH and C, TH and F, TL and C, and TL and F (*p* < 0.001 in all cases).

**Figure 2:**
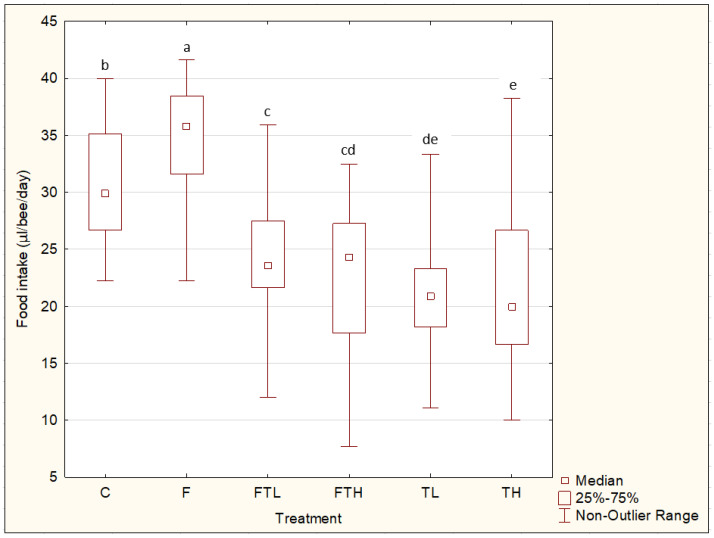
The daily average of the food intake per 1 bee over 14 days. Significant differences (*p* < 0.05) in food consumption between treatments are indicated by different letters.

The food intake was dependent on the treatment (Figure 2). The amount of diet consumed was higher in groups C and F than in any of the other groups, whereas the food consumption was higher in group F than in group C. In group FTL, higher food intake was observed than in group TL. The same trend was observed in the case of the FTH and TH groups. The lowest food consumption was observed in groups TH and TL.

**Figure 3:**
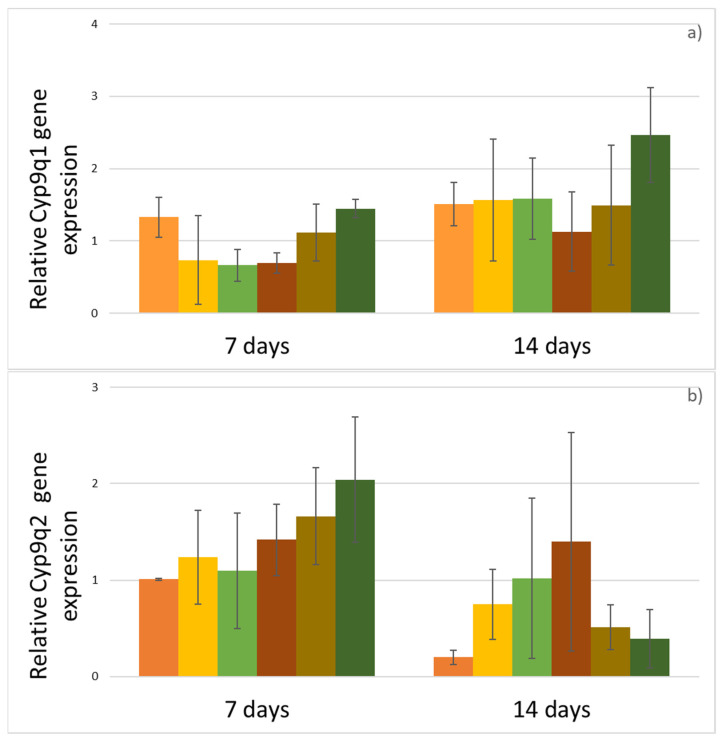

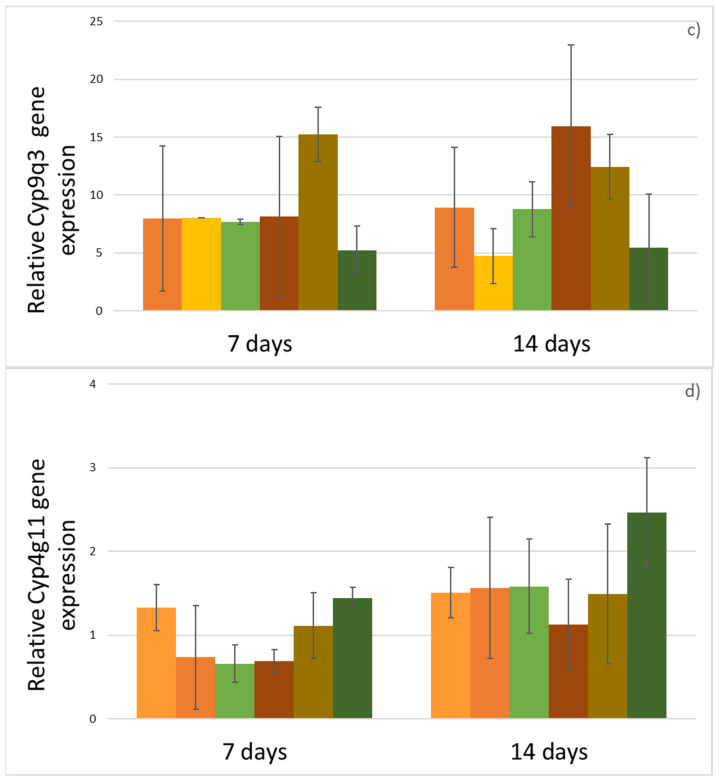
Relative expressions of four cytochrome P450 genes in bee workers depending on treatment at the 7th and 14th days of the experiment. Error bars denote two technical replications of three samples. (**a**) CYP9Q1 gene, (**b**)CYP9Q2 gene, (**c**) CYP9Q3 gene, (**d**) CYP4G11 genes.

The expression of CYP9Q1 (Figure 3a), CYP9Q2 (Figure 3b), CYP9Q3 (Figure 3c) and CYP4G11 (Figure 3d) genes was analyzed using RT-qPCR. Differences in gene expression between the testing groups were not statistically significant. However, despite that, some trends in the levels of expression were noted between the testing groups. The relative expression of the CYP9Q1 gene decreased in bees fed with sucrose solution enriched by phenolic compounds, irrespective of thiacloprid intoxification (F, FTL, FTH) in comparison with the C group after 7 days of treatment. In bees from groups TL and TH, the relative gene expression of this gene was comparable with its expression in the C group after 7 days. After 14 days of treatment, the relative expression of CYP9Q1 was increased in bees from group TH. In other groups, the relative gene expression of this gene was comparable with its expression in the C group.

After 7 days of treatment, the relative expression of CYP9Q2 in bees from groups F and FTL was comparable with the C group. In groups FTH, TL and TH, it was slightly increased in comparison with the C group. After 14 days of treatment, the relative expression of this gene was increased in groups F, FTL and FTH. In groups TH and TL it was comparable with group C.

The relative expression of CYP9Q3 was higher in the TL group after 7 days of treatment and also in the FTH and TL groups after 14 days of treatment. In other groups, the relative expression of this gene was comparable with group C.

After 7 days of treatment, the relative expression of the CYP4G11 gene was comparable in bees fed with sucrose solution enriched by phenolic compounds regardless of whether they were intoxicated with thiacloprid (F, FTL, FTH) and in bees from C group. In groups TL and TH, it was increased in comparison with C. After 14 days of treatment, the relative expression of this gene was increased in the FTH, TL and TH groups in comparison to the C group. In the F and FTL groups it was comparable with the C group.

## 4. Discussion

As expected, significantly higher mortality was observed in the treatment groups containing thiaclopride (TL, TH) compared to thiacloprid-free groups (C, F). This is consistent with the findings reported by Retschnig et al. [19]. However, Retschnig et al. [19] observed significantly lower mortality levels in bees intoxicated with high doses of thiacloprid than those observed in this study (TH). This difference may be due to different levels of sensitivity between bee subtaxons [20]. On the other hand, a low level of bee mortality was observed in group F, which did not differ from group C, indicating the safety of phenolic compounds for bees, which is in accordance with the results of Liao et al. [31]. Conversely, a statistically significant decrease was observed in the mortality rate of the group FTL in comparison with group TL, as well as in FTH compared to TH, which was probably caused by the increased detoxification capacity [30] and antioxidant activity [33] of the experimental bees due to phenolic-enriched diets [31]. The fact that with the addition of phenolic compounds (FTL), the mortality of intoxicated bees decreased significantly compared to the TL group, but did not reach the same level as in non-intoxicated bees (C), indicates the limited detoxification capacity in honey bees [28,42]. This trend was even more pronounced in the groups with a high dose of thiacloprid. A very significant reduction in mortality was observed with the addition of phenolic compounds (FTH) in comparison with the group without phenolic compounds (TH), but losses were still higher than in all other groups. The relationship between the experimental groups FTL, F and C seems to be an interesting phenomenon. No statistically significant difference in mortality was observed between the FTL and F groups, but there was a difference between the FTL and C groups. This can be explained by the probable increase of metabolic load caused by increased flavonoid levels [43].

The food consumption in the group containing phenolic compounds (F) was higher than in the group fed only with sucrose solution (C). Similar results were obtained by Porrini et al. [44]. They observed increased food intake when feeding bees with the addition of essential oils that contained phenolic compounds as their main components. Nevertheless, in their study, the rate of food consumption was lower in the control group, as well as in the experimental groups, compared to our study. This difference was probably caused by the difference in the carbohydrate concentration of the feed solution. A higher concentration of sugars in the feed leads to lower feed consumption, and vice versa [45]. On the other hand, in groups C and F, the food consumption rate was significantly higher than the groups with the addition of pesticide (TL and TH). This is consistent with the findings of Gregorc et al. [46] and Tosi et al. [47]. This was probably caused by increased levels of stress as a result of the addition of pesticides in the food [45]. However, in the case of intoxicated groups fed food enriched with phenolic compounds, the rate of food intake was significantly increased compared with groups without phenolic compounds, both in the case of low amounts of pesticide (FTL) and the case of high amounts of pesticide (FTH). This trend may be explained by the increased detoxification capacity [30] and antioxidant activity [33] caused by phenolic compounds in the food [31]. Differences in food consumption between TL and TH were not observed, nor were they observed between FTL and FTH. Therefore, the amount of pesticide in the food did not affect the level of consumption, which is consistent with the results of Retschnig et al. [19].

The relative expression of CYP9Q1, CYP9Q2, CYP9Q3 and CYP4G11 genes was analyzed using RT-qPCR. The cytochrome P450 enzyme group was chosen as the main endpoint in the detoxification process because it is responsible for the activity of the detoxification pathways of neonicotinoids [17]. The gene expression of four genes responsible for detoxication was analyzed after 7 and 14 days of treatment. In previous studies [30,37,48], bees were fed once with pesticide at the beginning of the experiment, and then the mortality and gene expression were analyzed in the first days after treatment. Conversely, in this study, a long-term experiment with long-term exposition to the tested substances was performed, with bees being fed continuously throughout the whole experiment. The expression levels of detoxification genes are highly dependent on time after pesticide treatment [49]. Therefore, the time of collection of genetic material could be the main reason why differences in detoxification gene expressions between experimental groups were not conclusive, and that the expression levels did not differ significantly between groups. Our results suggest a trend in which the expression in the F, FTL and C groups was comparable, and the gene expression in other intoxicated groups was increased. This could indicate that increased expression probably took place at the beginning of experiment, and that in the first days of bee sampling, the level of enzymes cytochrome P450 were already increased. However, better explanation of this issue could be provided by quantification of expressed protein. It would be suitable to carry out this investigation in future experiments.

Wheeler and Robinson [50] point out the problem that beekeepers use artificial bee food for bees, which, however, usually does not contain certain ingredients with high nutritional value and importance that are natural components of honey and pollen. Thus, it is clear that in addition to macronutrients (carbohydrates and proteins), the bee diet should also contain other elements (such as phenolic compounds) that have a conclusive impact on their detoxification capacity [30,31]. Based on the results of this study, we suggest that the addition of phenolic compounds to bee nutrition could to some extent increase the detoxification capacity of bees [30,31], which is often reduced due to malnutrition caused by degradation of the environment and the associated loss and contamination of food resources, as well as factors related to routine beekeeping management [4,50]. In addition, according to Mao et al. [29], some phenolic substances have an effect on the suppression of ovarian development, suggesting that phenolic substances could be used in the future to solve other problems in beekeeping practice.

## 5. Conclusions

Phenolic compounds, as natural components of the bee diet, have been demonstrated to have a positive impact on the longevity of honey bees intoxicated by thiacloprid, as well as their food intake.

The results of the experiments suggest that by adding phenolic substances to bee nutrition, the risks associated with the intoxication of bees can be reduced.

The expression levels of detoxification genes alone, depending on the treatment, may not be sufficient, and it is appropriate to support this with quantification of expressed proteins.

## Figures and Tables

**Table 1:** Content of phenolic acids and flavonoids used in the phenolic mixture.

Phenolic Substance Classification	Phenolic Substance Name	Amount (%)	Amount (mg/kg)
Phenolic acids	caffeic acid	10	20
	benzoic acid	20	40
	gallic acid	7.5	15
	ferulic acid	20	40
	p-Coumaric acid	35	70
	vanillic acid	7.5	15
Flavonoids	rutin	25	2.5
	quercetin	25	2.5
	naringin	25	2.5
	hesperidin	25	2.5

**Table 2:** Primers for qPCR analysis.

Gene	Sequences 5′-3′	Reference
Cyp9q1	F: TCGAGAAGTTTTTCCACCG R: CTCTTTCCTCCTCGATTG	Mao et al. [37]
Cyp9q2	F: GATTATCGCCTATTATTA R: GTTCTCCTTCCCTCTGAT	Mao et al. [37]
Cyp4g11	F: AATGCGAGAAGTGTCGTCGA R: AGCGGTTTCCAGAAGGATGT	Calla et al. [38]
AmRp49	F: CGTCATATGTTGCCAACTGGT R: TTGAGCACGTTCAACAATGG	Tesovnik et al. [39]

**Table 3:** The results of the log-rank test, which was used to compare different treatment groups of bees treated using various chemical substances.

Treatment	Degrees of Freedom	Chi-Square Statistic	*p*-Value
TH/C	1	310	<0.001
TH/F	1	270	<0.001
FTH/TH	1	72	<0.001
FTH/C	1	62.9	<0.001
FTH/F	1	51.2	<0.001
FTL/TL	1	6	0.01
TL/C	1	5.7	0.01
FTL/C	1	4.6	0.03
FTL/F	1	1.8	0.17
F/C	1	0.6	0.44

## Data Availability

The study did not report any data.
